# CREATING DIVERSE SPACES IN UNDERGRADUATE EDUCATION: OLDER LGBT RESEARCH AND TEACHING

**DOI:** 10.1093/geroni/igz038.1316

**Published:** 2019-11-08

**Authors:** Maggie Syme

**Affiliations:** Kansas State University, Manhattan, Kansas, United States

## Abstract

According to SAGE (Services and Advocacy for GLBT Elders), an estimated 3 million older adults currently identity as lesbian, gay, bisexual, or transgendered (LGBT), which is expected to double in the next decade, making them one of the fastest growing minority aging groups. Yet, there is very little attention being paid to older LGBT individuals, a dearth of information on their experiences, and multiple stigmas that compound to make this a challenging space for education and research. This presentation will discuss several different strategies to integrate research and teaching in order to make LGBT elders a relevant and impactful topic in undergraduate education. These include students undertaking field research on dating ads, creating infographics for health challenges, and evaluating sexual wellness research to illustrate similarities and differences among LGBT older adults and their heterosexual counterparts. Specific considerations for approaching topics in undergraduate contexts will be discussed.

